# Molecular profiling of *ETS* and non‐*ETS* aberrations in prostate cancer patients from northern India

**DOI:** 10.1002/pros.22989

**Published:** 2015-03-23

**Authors:** Bushra Ateeq, Lakshmi P. Kunju, Shannon L. Carskadon, Swaroop K. Pandey, Geetika Singh, Immanuel Pradeep, Vini Tandon, Atin Singhai, Apul Goel, Sonal Amit, Asha Agarwal, Amit K. Dinda, Amlesh Seth, Alexander Tsodikov, Arul M. Chinnaiyan, Nallasivam Palanisamy

**Affiliations:** ^1^ Department of Biological Sciences and BioengineeringIndian Institute of TechnologyKanpurIndia; ^2^ Michigan Center for Translational PathologyUniversity of Michigan Medical SchoolAnn ArborMichigan; ^3^ Department of PathologyUniversity of MichiganUniversity of Michigan Medical SchoolAnn ArborMichigan; ^4^ Comprehensive Cancer CenterUniversity of Michigan Medical SchoolAnn ArborMichigan; ^5^ Department of PathologyAll India Institute of Medical SciencesNew DelhiIndia; ^6^ Digdarshika Pathology LaboratoryLucknowIndia; ^7^ Department of PathologyKing George's Medical UniversityLucknowIndia; ^8^ Department of UrologyKing George's Medical UniversityLucknowIndia; ^9^ Department of PathologyGSVM Medical CollegeKanpurIndia; ^10^ Department of UrologyAll India Institute of Medical SciencesNew DelhiIndia; ^11^ Department of BiostatisticsUniversity of MichiganAnn ArborMichigan; ^12^ Howard Hughes Medical InstituteUniversity of Michigan Medical SchoolAnn ArborMichigan; ^13^ Department of UrologyHenry Ford Health SystemDetroitMichigan

**Keywords:** genetic rearrangements, *TMPRSS2‐ERG*, *SPINK1*, *RAF* kinase, *PTEN*

## Abstract

**BACKGROUND:**

Molecular stratification of prostate cancer (PCa) based on genetic aberrations including *ETS* or *RAF* gene‐rearrangements, *PTEN* deletion, and SPINK1 over‐expression show clear prognostic and diagnostic utility. Gene rearrangements involving *ETS* transcription factors are frequent pathogenetic somatic events observed in PCa. Incidence of *ETS* rearrangements in Caucasian PCa patients has been reported, however, occurrence in Indian population is largely unknown. The aim of this study was to determine the prevalence of the *ETS* and *RAF* kinase gene rearrangements, SPINK1 over‐expression, and *PTEN* deletion in this cohort.

**METHODS:**

In this multi‐center study, formalin‐fixed paraffin embedded (FFPE) PCa specimens (n = 121) were procured from four major medical institutions in India. The tissues were sectioned and molecular profiling was done using immunohistochemistry (IHC), RNA in situ hybridization (RNA‐ISH) and/or fluorescence in situ hybridization (FISH).

**RESULTS:**

*ERG* over*‐*expression was detected in 48.9% (46/94) PCa specimens by IHC, which was confirmed in a subset of cases by FISH. Among other *ETS* family members, while *ETV1* transcript was detected in one case by RNA‐ISH, no alteration in *ETV4* was observed. SPINK1 over‐expression was observed in 12.5% (12/96) and *PTEN* deletion in 21.52% (17/79) of the total PCa cases. Interestingly, *PTEN* deletion was found in 30% of the ERG‐positive cases (*P* = 0.017) but in only one case with SPINK1 over‐expression (*P* = 0.67). *BRAF* and *RAF1* gene rearrangements were detected in ∼1% and ∼4.5% of the PCa cases, respectively.

**CONCLUSIONS:**

This is the first report on comprehensive molecular profiling of the major spectrum of the causal aberrations in Indian men with PCa. Our findings suggest that *ETS* gene rearrangement and SPINK1 over‐expression patterns in North Indian population largely resembled those observed in Caucasian population but differed from Japanese and Chinese PCa patients. The molecular profiling data presented in this study could help in clinical decision‐making for the pursuit of surgery, diagnosis, and in selection of therapeutic intervention. *Prostate 75:1051–1062, 2015*. © 2015 The Authors. *The Prostate*, published by Wiley Periodicals, Inc.

## INTRODUCTION

Prostate cancer (PCa) is one of the most common malignancies after lung cancer and the second most common cause of cancer‐related death among men worldwide. The incidence of PCa in India has been steadily increasing concomitant with an increase in life expectancy. According to the National Cancer Registry program by the Indian Council of Medical Research, New Delhi, PCa is estimated to increase by 140% in the next few years 1, 2. The challenges posed in diagnosing and treating prostate cancer is attributed to the clinical and molecular heterogeneity associated with this disease. The seminal discovery of the recurrent (>50%) genetic rearrangements involving the androgen‐regulated gene transmembrane protease, serine 2 (*TMPRSS2*), with v‐ets erythroblastosis virus E26 oncogene homolog (avian) (*ERG*) in PCa prompted the molecular categorization of PCa into distinct molecular subtypes 3, similar to hematologic malignancies, for identifying patients with aggressive subtypes and distinct therapeutic targets. Subsequently, discovery of genetic rearrangements involving *TMPRSS2* with other 3' erythroblastosis virus E26 transformation‐specific (*ETS*) transcription factor family members such as *ETV1* (∼5%), *ETV4* (∼5%), or *ETV5* (∼1%) led to further classification of PCa.

In a separate study using Cancer Outlier Profile Analysis (COPA), SPINK1 (serine peptidase inhibitor, Kazal type 1) was shown to be over‐expressed in ∼10–15% of the total PCa cases 4. Interestingly, SPINK1 and ETS over‐expression demonstrated mutually exclusive pattern across multiple independent PCa cohorts, and *SPINK1* outlier expression was associated with an aggressive subset of prostate cancers 4, 5, 6.

Another important molecular event in PCa is the inactivation of the phosphatase and tensin homolog (*PTEN*) gene by genomic deletion or rearrangement, including intragenic breakage and translocation. *PTEN* deletion or rearrangement has been reported in 20–30% of PCa and is also associated with aggressive cancers 7, 8, 9. Interestingly, mouse model of PCa demonstrated that *PTEN* loss and *ERG* genetic rearrangement might cooperate in the development of prostate adenocarcinoma 10, 11. Many independent studies have shown that *PTEN* deletion and *ERG* genetic alterations frequently coexist, and incidences of *PTEN* deletions are more frequent in *ETS* fusion‐positive PCa than in fusion‐negative cancers 7, 8, 12, 13.

Using paired‐end next‐generation sequencing approach, the druggable RAF kinase genetic rearrangements, *SLC45A3‐BRAF* (solute carrier family 45, member 3–v‐raf murine sarcoma viral oncogene homolog B1) and *ESRP1‐RAF1* (epithelial splicing regulatory protein‐1–v‐raf‐1murine leukemia viral oncogene homolog‐1) were discovered in ∼2% of the PCa patients 14. Most importantly, the *RAF* genetic rearrangements demonstrated sensitivity to sorafenib, a US Food and Drug Administration (FDA) approved RAF/MEK inhibitor suggesting that *RAF* genetic rearrangements, although rare, may benefit patients positive for *RAF* rearrangements 14.

Although *TMPRSS2‐ERG* gene fusion is one of the highly recurrent (∼50%) oncogenic drivers in PCa, it has been a challenging therapeutic target. A recent study demonstrated a potential strategy to therapeutically target ERG via its interacting partner protein, poly‐ADP‐ribose polymerase (PARP) for the treatment of *ERG* fusion‐positive PCa 15. Interestingly, lower incidence of *ERG* genetic rearrangement has been reported in African Americans (∼28%) in comparison to ∼50–60% recurrence in Caucasian Americans. Moreover, incidence of *PTEN* deletion is low (7%) in African American men compared to Caucasian men and the prevalence of SPINK1 over‐expression is high (∼23%) among African American men compared to their Caucasian counterpart 16. Much lower recurrence of *ERG* alteration (∼10–20%) has been reported among the Japanese 17 and Chinese PCa cohort 18 compared to the Western population. Moreover, Filipinos with PCa are at increased risk of progressing to advanced stages of PCa and have lower survival rates compared to other Asians. Prevalence of *TMPRSS2‐ERG* rearrangement is 23% among Filipino PCa patients with an increased rate (∼33%) observed in the advanced PCa cohort 19. Comprehensive landscape of genetic alterations in PCa patients from the Indian sub‐continent is currently lacking. Recently, a report on PCa patients from India reported low recurrence (∼27%) of *TMPRSS2‐ERG* genetic rearrangement 20. However, inherent limitations of this study include the small cohort size (n = 30) and all samples analyzed were from a single institution, thus extrapolation of these results to broader population is difficult. Here, to study the genetic heterogeneity of PCa in the Indian population, we performed comprehensive characterization of patient samples to determine the prevalence of genetic rearrangements involving *ETS* gene family members (*ERG*, *ETV1*, and *ETV4*); non‐*ETS* genetic rearrangements such as *RAF* kinase family fusions (*BRAF* or *RAF1*), SPINK1 over‐expression and the status of the tumor suppressor gene *PTEN*. Our data revealed that the *ETS* gene rearrangement and SPINK1 over‐expression patterns in the North Indian population largely resembled those observed in the Caucasian population but differed from Japanese and Chinese PCa patients.

## MATERIALS AND METHODS

### Prostate Specimens

A total of 121 PCa specimens (including five benign prostate specimens) were procured from All India Institute of Medical Sciences, New Delhi; King George's Medical University, Lucknow; Digdarshika Pathology Laboratory, Lucknow; and GSVM Medical College, Kanpur. A total of 22 PCa specimens were excluded from the study based on low tumor content and integrity of the cancer tissues. All PCa specimens were collected from patients with written informed consent and upon Institutional Review Board approval. The study PCa cohort included specimens from men who underwent prostatectomy (n = 40), needle core biopsies (n = 48), and transurethral resection of the prostate (TURP) (n = 33) to relieve obstructive symptoms from locally advanced disease at the participating institutions between year 2010 and 2014. None of the patients received preoperative radiation or androgen deprivation therapy. All patients included in this study were of Indian descent residing in northern India. All specimen included in the study were de‐identified.

### ERG and SPINK1 Immunohistochemistry

Immunohistochemistry was performed on unstained formalin‐fixed, paraffin‐embedded (FFPE) tissue sections obtained from the prostatectomy, needle core biopsies, or TURP specimen blocks. Primary monoclonal antibody against ERG, clone *EPR* 3864 (Epitomics, Burlingame, CA) or monoclonal antibody against SPINK1 (H00006690‐M01‐Abnova, Taiwan) was used on the automated Ventana Benchmark staining platform (Ventana Medical Systems‐A Roche group, Tucson, AZ). Staining of blood vessels was used as a positive control and slides displaying no staining of vessels were excluded from further analysis. All IHC slides were evaluated by a board certified pathologist (LPK).

### Fluorescence In Situ Hybridization (FISH)

FISH assays were performed on unstained FFPE sections obtained from the PCa specimen blocks. BAC clones (*ERG* 5'‐RP11‐830D9; *ERG* 3'‐RP11‐143L14; *PTEN* locus probe‐RP11‐165M8; chr10 control probe RP11‐351D16; *ETV4* 5'‐RP11‐147C10; *ETV4* 3'‐CTD3215I16; *BRAF* 5'‐RP11‐767F15; *BRAF* 3'‐RP11‐248P7; *RAF1* 5'‐RP11‐767C1; *RAF1* 3'‐RP11‐586C12) were selected from the UCSC genome browser and purchased through BACPAC resources (Children's Hospital, Oakland, CA). Following colony purification DNA was prepared using QiagenTips‐100 (Qiagen, Valencia, CA). DNA was labeled by nick translation method with biotin‐16‐dUTP and digoxigenin‐11‐dUTP for 3' and 5' probes and *PTEN* locus and CEN10 control probes respectively (Roche, USA). Probe DNA was precipitated and dissolved in hybridization mixture containing 50% formamide, 2X SSC, 10% dextran sulphate, and 1% Denhardt's solution. Approximately two hundred nanogram of labeled probe was hybridized to normal human chromosomes to confirm the map position of each BAC clone. FISH signals were obtained using anti‐digoxigenin‐fluorescein and AlexaFluor‐594 conjugate to obtain green and red colors, respectively. Fluorescence images were captured using a high resolution CCD camera controlled by ISIS image processing software (Metasystems, Germany).

Deletion of *PTEN* was defined as fewer than two copies of the gene specific probe in the presence of two reference signals in >20% of the tumor nuclei. For both *ERG* rearrangement and *PTEN* deletion, at least one hundred tumor nuclei per case were evaluated under a fluorescence microscope (Carl Zeiss & Metasystems, Germany).

### RNA In Situ Hybridization and Evaluation Criteria

RNA in situ hybridization (RNA‐ISH) was performed as described previously using RNAscope FFPE Reagent Kit 2.0 (Advanced Cell Diagnostics, Hayward, CA) 21. Briefly, FFPE sections were baked at 60 °C for 1 hr. *ETV1* RNA probe (*ETV1* Accession Number NM_004956.4, region 998‐2031) and POLR2A (as a positive control) were designed by Advanced Cell Diagnostics (Hayward, CA). Tissues were deparaffinized in xylene twice for 15 min each with periodic agitation, slides were then immersed in 100% ethanol twice for 3 min each with periodic agitation and air‐dried for 5 min. Tissues were circled using a pap pen (Vector, H‐4000), allowed to dry and treated with pretreatment 1 buffer for 10 min. The slides were processed using previously established protocol 21 and mounted in Cytoseal XYL (Thermo Scientific, #8312‐4) for viewing under bright‐field microscope. Positive controls were performed for all runs using a POLR2A gene‐specific RNA probe.

RNA‐ISH expression intensity scoring guidelines were established to classify tumor foci as *ETV1* positive or *ETV1* negative 22. *ETV1* expression by RNA‐ISH appeared as distinct cytoplasmic punctate dots. All tumor foci were evaluated and scanned at 20× magnification. Scoring for an entire tumor focus was based on the highest *ETV1* intensity using criteria previously reported 21. All *ETV1* RNA‐ISH slides were reviewed by study pathologist (LPK).

### Statistical Analysis

Fisher's exact tests were used to evaluate association between categorical variables. For analyzing the association between *PTEN* deletion status (response, normal vs. deletion) with co‐occurrence of *ERG* or *SPINK1* positive status logistic regression has been applied. For assessing any association between *ERG* positive, *SPINK1* positive, or *SPINK1* negative statuses and Gleason score (using numeric Gleason score as a response) linear regression model was used. For all statistical tests, a *P* < 0.05 was considered statistically significant.

## RESULTS

### Prevalence of *ERG*, *ETV1*, and *ETV4* Genetic Rearrangements

**Figure 1:**
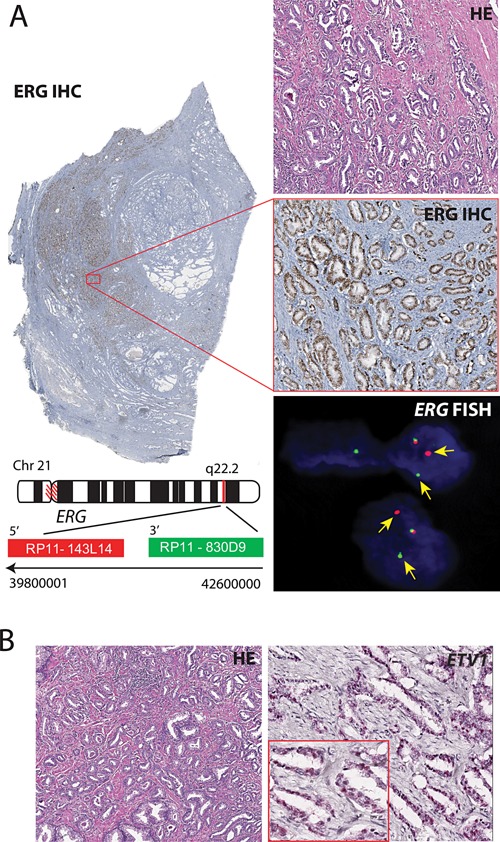
IHC staining and FISH for *ERG* rearrangement and RNA‐ISH for *ETV1*. Panel **A**, whole section view of a prostate carcinoma tissue (Gleason score 3 + 3) showing ERG expression by IHC staining (panel on left). Right panel showing IHC staining for ERG over‐expression (×20 magnification) with the corresponding H&E stained sections (top). Bottom panel shows FISH for *ERG* genetic rearrangement using *ERG* break‐apart probes. Yellow arrow shows rearranged *ERG*, and co‐localizing red‐green signal depicts intact *ERG* locus. Panel **B** shows *ETV1* expression by RNA‐ISH with a maximum intensity score 4 (×20 magnification).

ERG oncoprotein expression was evaluated by IHC on 94 PCa biopsies and five benign prostate specimens. A total of 46 out of 94 PCa (48.9%) were found to be positive for ERG expression (Fig. 1A) and all five benign PCa specimens were negative for ERG over‐expression. We next confirmed *ERG* gene rearrangement in a subset of patients (n = 17) that were positive for *ERG* staining by FISH. As anticipated, all cases showed *ERG* rearrangement either by *ERG* split signal or 5' deletion of *ERG* (Fig. 1A, bottom panel). Results from the FISH assay of *ERG* rearrangement‐positive cases revealed that *ERG* gene fusion occurred in neoplastic cells but not in adjacent benign nuclei or stromal cells. The majority of the cases included in this study had index tumor Gleason scores of 7 or higher (82/94, 87%) and all available clinical information associated with the patients' specimens are listed in Supplementary Table 1. For the remaining 43 *ERG*‐negative cases, *ETV1*, and *ETV4* rearrangement detection was performed by RNA‐ISH and FISH, respectively. None of the 43 (0/43) PCa specimens tested by *ETV4* break‐apart FISH were positive and only one PCa specimen was detected as *ETV1* positive as evaluated by RNA‐ISH (Fig. 1B). Of note, the *ETV1*‐positive case was negative for ERG and SPINK1 over‐expression by IHC. None of the benign specimens (n = 5) were positive for *ERG*, *ETV1*, or *ETV4* genetic rearrangements.

### Prevalence of SPINK1 Over‐Expression

**Figure 2:**
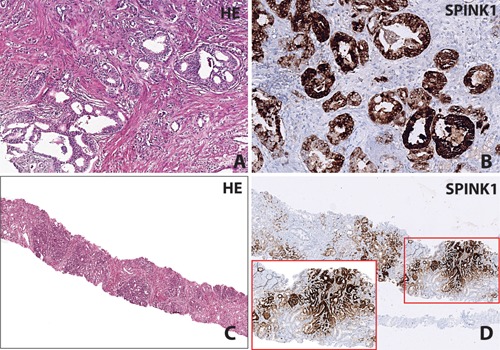
IHC staining for SPINK1. Panel **A** and **B** show a prostatic adenocarcinoma with SPINK1 positive immunostaining with the corresponding H&E stained section; Gleason score 3 + 4 (×20 magnification). Panel **C** and **D** show a needle core biopsy which demonstrates SPINK1 positive immunostaining with the corresponding H&E stained section; Gleason score 3 + 3 (×4 and ×10 magnification in inset).

SPINK1 over‐expression was evaluated by performing IHC on all the ERG*‐*negative cases and the cases where the ERG‐positive immunostaining was heterogeneous (7 cases). A total of 66 PCa specimens were immunostained for the SPINK1 over‐expression, 12 cases were confirmed as SPINK1‐positive representing 12.76% of the 94 total PCa specimens (Fig. 2A–D). Importantly, none of the SPINK1‐positive cases were *ERG* rearrangement‐positive, confirming mutual exclusivity of both genetic alterations. Moreover, all seven ERG‐positive heterogeneous cases were found to be negative for SPINK1. Of note, *SPINK1* prevalence in our cohort is similar to the *SPINK1* incidence (∼10–15%) reported in the Caucasian cohorts 4, 5. As anticipated none of the benign specimen (n = 5) were positive for SPINK1 over‐expression. No significant association between *ERG* positive status and Gleason score was found when adjusted for *SPINK1* status (*P* = 0.42) using a linear model (numeric Gleason scores) and an ordinal proportional odds regression with fine ordinal categorization of Gleason scoring (<6, 3 + 4, 4 + 3, 8–10). Although SPINK1 negative cases showed a trend for increased Gleason score (0.58, SE = 0.29, *P* = 0.053) using a linear regression model. The effect reached significance in the ordinal model with the cumulative logOR of 1.26 (SE = 0.64, *P* = 0.049).

### Concomitant Presence of *ERG* Rearrangement, SPINK1 Over‐Expression, and *PTEN* Deletion

**Figure 3:**
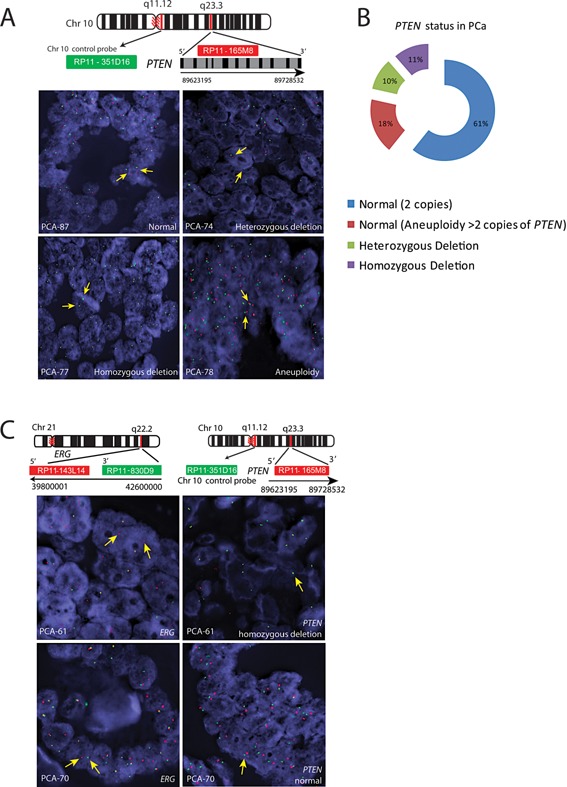
FISH for *PTEN* aberrations. Panel **A**, schematic diagram showing the genomic organization of *PTEN* gene on Chr10 q23.3 and chromosome 10 control probe (q11.12). Green and red bars indicate the chromosome 10 control probe and *PTEN* BAC clones, respectively. FISH images of the PCa specimens, PCA‐87 shows normal *PTEN* copy number with two red and two green signals corresponding to *PTEN* and Chr10 control probe, respectively. PCA‐74 shows heterozygous deletion of *PTEN* showing loss of one red signal, but two green centromere signals. PCA‐77 depicts homozygous deletion of *PTEN* (loss of red signals), but two green centromere signals. PCA‐78 shows aneuploidy with 3‐4 copies of chromosome 10 and *PTEN*. Panel **B**, percent distribution of the *PTEN* aberrations. Panel **C** shows *ERG* genetic rearrangement using *ERG* break‐apart probes and homozygous deletion of *PTEN* in the same patient (PCA‐61). Likewise, PCA‐70 shows *ERG* genetic rearrangement and normal *PTEN* status.

To assess *PTEN* genomic deletion, a two color interphase FISH approach was used as *PTEN* deletions are interstitial and are usually limited to small regions of chromosome 10. The *PTEN* FISH was performed on 92 PCa specimens, of those 13 specimens were excluded from the analysis due to no signal or no hybridization on the tissue sections. Homozygous deletion of *PTEN* was observed in 11.39% (9 out of 79) of the PCa cases whereas hemizygous deletion was observed in 10.13% (8 out of 79) of the PCa cases (Fig. 3A–C). We also observed aneuploidy (>2 copies of *PTEN*) in 17.72% (14 out of 79) of the PCa cases (Fig. 3A–B). Interestingly, 30% (14 out of 46) of the ERG‐positive cases showed *PTEN* deletion compared to only one SPINK1‐positive case (1 out of 12) with *PTEN* deletion. Logistic regression was applied to model the association of the *PTEN* deletion status (response, normal vs. deletion) with ERG and SPINK1 positive status. *ERG* positivity leads to an increase in the chances to develop a *PTEN* deletion of 1.65 on the logit scale (SE = 0.69, *P* = 0.017). Conversely, the association of SPINK1 positive status with *PTEN* deletion was non‐significant (*P* = 0.67), and point estimate of the effect was much smaller (0.64 on logit scale, SE = 1.47). Thus, our findings corroborate with the previous studies demonstrating a significant overlap between *ERG* genetic rearrangements and *PTEN* deletions 12, 23. Moreover, the incidence of ERG positivity in patients with *PTEN* deletions was 0.82, exact 95% Confidence Interval (CI) (0.59, 0.94). Likewise the incidence of SPINK1 positive status with *PTEN* deletion was much smaller, 0.33, exact 95% CI (0.008, 0.91), although the sample size for the latter is too small to be interpreted.

### Incidence of *BRAF* and *RAF1* Rearrangements

**Figure 4:**
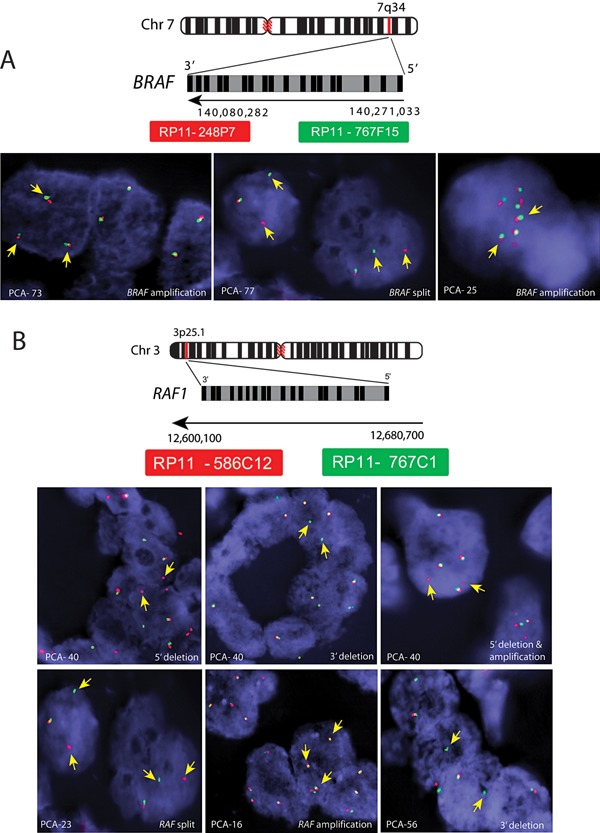
FISH for *RAF* genetic rearrangements. Panel **A**, schematic diagram showing the genomic organization of *BRAF* gene on chr7q34. Green and red bars indicate the 5' and 3' BAC clones, respectively. FISH images of the PCa specimens (PCA‐73 & 25) show *BRAF* amplification (left) and *BRAF* rearrangement (middle) in PCA‐77 (right). Panel **B**, schematic diagram showing the genomic organization of the *RAF1* gene on chr3p25. Green and red bars indicate 5' and 3' BAC clones, respectively. FISH images of the PCa specimens (PCA‐40) show *RAF1* rearrangement with 5' deletion (green signal missing), 3' deletion (red signaling missing), and 5' deletion with 3 copies of normal *RAF1* in the top panel. Likewise, PCa specimens PCA‐23, PCA‐16, and PCA‐56 show *RAF* rearrangement, amplification, and 3' deletion, respectively (bottom panel).

*BRAF* and *RAF1* rearrangements were detected by FISH using break‐apart probes on 88 and 63 PCa specimens, respectively. Interestingly, one out of 88 (∼1%) PCa cases harbored *BRAF* genetic rearrangement and two cases displayed focal amplification with 5–7 copies of *BRAF* (Fig. 4A). These results are consistent with our previous finding that reported ∼2% incidence of *BRAF* rearrangement in prostate cancer 14. *RAF1* rearrangement was observed in ∼4.5% (3 out of 66) of PCa cases; PCA‐23 harbored *RAF1* rearrangement, second case (PCA‐56) displayed 3' deletion and third case (PCA‐40) displayed 3' deletion in one tumor foci and *RAF* amplification and 5' deletion in another tumor foci (Fig. 4B, top panel). We also observed a single case (PCA‐16) with 4 copies of *RAF1* resulting from aneuploidy (Fig. 4B, bottom panel). Notably, all the cases that were positive for rearrangements or amplification of *BRAF* or *RAF1* had features of advanced PCa including high Gleason score (four cases with Gleason score 9 and two cases with Gleason score 7). Intriguingly, all the *RAF* rearrangement‐positive cases were also positive for *ERG* rearrangement, but not the sample harboring *BRAF* amplification.

## DISCUSSION

In this study, we stratified Indian PCa cohort based on the molecular alterations known to be prevalent among different PCa populations in the world 3, 4, 24, 25, 26, 27. To our knowledge, this is the first study to comprehensively evaluate the entire spectrum of driving aberrations in Indian PCa samples that include *ERG*, *ETV1, ETV4, RAF* kinase genetic rearrangements, SPINK1 over‐expression, and *PTEN* deletion. Our findings indicate a higher incidence of *ERG* rearrangement (49%) in the Indian PCa cohort compared to a recent report by Rawal and colleagues (27%) 20. This discrepancy could arise from the smaller cohort size (total 30 PCa cases) and the samples collected from a single medical institution. Additionally, they characterized only the *ERG* gene rearrangement using a different ERG antibody (9FY clone, Biocare Medical Inc.) 20 for IHC analysis as well as variation in evaluation criteria, and possibly therapeutic interventions (anti‐androgen therapy). Most importantly, Park *et al*., demonstrated that ERG immunostaining using same monoclonal antibody that we have used against ERG had an overall 95.7% sensitivity and 96.5% specificity for *ERG* rearrangements, suggesting that ERG expression by IHC has high concordance with FISH 28.

Although, *ETS* genetic rearrangement represents a highly prevalent genetic alteration (50–60%) among PCa patients of the Western world, therapeutic targeting of *ETS* transcription remains a challenge. Nevertheless, we have shown that ERG interacts with the enzyme poly (ADP‐ribose) polymerase 1 (PARP1) in a DNA‐independent manner and pharmacological inhibition of PARP1 inhibits *ETS*‐positive PCa xenograft 15. Our findings also showed that *ETV1* genetic rearrangements are less recurrent (∼1%) in Indian PCa cohort than what has been reported in the Caucasian cohorts (∼5%) 24, 29, although studies with larger patient cohorts are needed to validate our finding. Interestingly, Baena *et al*., demonstrated that ERG and ETV1 regulates androgen receptor (AR) target genes inversely, ERG negatively regulates the AR transcriptional program whereas ETV1 enhances AR signaling and activation of the AR transcriptional program 30. Our data corroborates previous findings that *ERG* rearrangements are clonal in nature as all cells in a given cancer foci were positive for the ERG expression, while distinct cancer foci in a single prostate may have differing *ERG* rearrangement status 12, 31, 32, 33. However recently, existence of rare molecular subsets of PCa with dual gene rearrangements, such as ERG/SPINK1 34, *ERG/ETV1*, and *ERG*/*ETV4* in different tumor focus of the same tumor has been noted 22.

Similar to the incidences reported in Caucasian PCa cohorts 4, 5, we also identified SPINK1 over‐expression in ∼12% of the PCa cases in Indian subcontinent. A recent report on African American men (n = 105) with PCa showed higher SPINK1 incidence (23.8%) compared to only ∼8% SPINK1‐positive cases in Caucasian PCa samples (n = 113) 16, highlighting differences at the molecular level in these two clinicopathologically matched PCa cohorts and racial disparities in prostate cancer 35, 36, 37. Several independent studies have confirmed the mutual exclusivity of *SPINK1* and *ERG* rearrangements in PCa 4. SPINK1 over‐expression is associated with aggressive phenotype among *ETS‐*negative PCa cases, with higher risk of biochemical recurrence than SPINK1‐negative patients 4, 5. Previously, we showed that SPINK1 interacts with epidermal growth factor receptor (EGFR) and activates downstream signaling and EGFR dimerization 38. Moreover, monoclonal antibodies to either SPINK1 or EGFR (cetuximab) significantly slowed down tumor growth in SPINK1‐positive tumor xenografted mice 38. Of note, multiplex assay of *SPINK1* and *TMPRSS2‐ERG* along with *GOLPH2* and *PCA3* transcript expression could be utilized as predictors of PCa, and most importantly these biomarkers could outperform serum PSA or *PCA3* alone in detecting the disease 39.

Deletions or mutations in *PTEN*, which encodes a phosphoinositide 3‐phosphatase, have been found to be associated with higher Gleason score, metastasis, hormone resistance, and an overall poor prognosis 9, 26, 40. Furthermore, the clinical significance of molecular aberrations in *PTEN*, *TMPRSS2‐ERG*, and *SPINK1* has been demonstrated in the development of castration‐resistant prostate cancer (CRPC) 23. Here, we found almost equal proportion of homozygous (11.39%) and heterozygous *PTEN* deletions (10.13%) in the Indian PCa cohort. A recent report demonstrated that heterozygous *PTEN* deletions are less frequent in African American PCa cohort (6.9%) than in Caucasian PCa cohort (19.8%) 16. Moreover, homozygous *PTEN* deletions are more prevalent in CRPC whereas *PTEN* heterozygous deletions occurred at higher frequency in localized PCa 6, 23. Importantly, loss of *PTEN* results in increased AKT and mTOR signaling, suggesting that alterations in the mTOR/AKT pathways could also be therapeutically targeted in patients harboring *PTEN* loss. Indeed, a recent study demonstrated promising therapeutic outcomes upon dual inhibition of AKT and mTORC1 in the preclinical genetically‐engineered mouse (GEM) model of CRPC 41.

In the present study, we found ∼4.5% (3 out of 66) *RAF1* genetic rearrangements and ∼1% (1 out of 88) *BRAF* genetic rearrangement in the Indian PCa cohort. Likewise, lower incidence of the *BRAF* (2.5%) and *RAF1* (1.5%) aberrations has been reported in the Chinese PCa cohort as well 42. Nevertheless, a high incidence of *BRAF* (29%) and *RAF1* (15%) copy number gain was observed in the same cohort, suggesting activation of the RAS/RAF/MEK/ERK signaling pathway 42. In contrast to Chinese PCa cohort, we found only one case with *BRAF* focal amplification; however more studies with larger cohort size are needed to confirm our finding. One of the limitations of our study is the lack of patients' follow‐up information and the evaluation of associations with clinical outcome, as follow‐up of the cancer patients is poor in India due to inadequate health/medical awareness and socioeconomic status of the patients. Nevertheless, the current study provides an initial molecular stratification of the PCa in this patient population that could aid in clinical decision‐making for the pursuit of surgical, targeted therapy, hormonal and/or chemo, and radiation therapy. Moreover, in the current genomic and precision therapy era, the diagnosis and treatment of PCa is rapidly evolving 43, 44. Hence, comprehensive molecular characterization of PCa patients from Indian sub‐continent utilizing high‐throughput sequencing approaches will inform the pursuit of appropriate targeted therapies.

In summary, this is the first comprehensive report demonstrating the prevalence of the *ETS* gene‐rearrangements, SPINK1 over‐expression, druggable *RAF* rearrangements and *PTEN* aberrations prevalent among Indian men with PCa. Taken together, Indian PCa population characterized in this study largely resembled the *ETS* gene rearrangement and SPINK1 over‐expression scenario observed in the Caucasian race, and differed from the prevalence reported in Japanese and Chinese patients; suggesting racial disparity and differences at the molecular level in prostate cancer. Most importantly, similar to *ALK* gene fusions (∼5% of the cases) in non‐small cell lung cancer patients, who benefit from ALK kinase inhibitors; the higher incidence of *RAF* rearrangements positive PCa patients (∼5% in Indian PCa) reported in this study may respond to the FDA‐approved RAF inhibitors or MEK inhibitors. Therefore, understanding the underlying common molecular driver alterations prevalent among Indian PCa patients will permit optimization of the screening methods and selection of the appropriate treatment regimen in this population.

## DISCLOSURE/CONFLICT OF INTEREST

N.P. receives research funding and honoraria from the Ventana/Roche but this study was not supported by this funding A.M.C. serves on the advisory board of the Gen‐Probe; and is a co‐inventor on a patent filed by the University of Michigan covering the diagnostic and therapeutic field of use for *ETS* fusions in prostate cancer. The Ventana/ Roche and Gen‐Probe did not play a role in the design and conduct of this study, in the collection, analysis, or interpretation of the data, or in the preparation, review, or approval of the article. The remaining authors declare no conflicts of interest.

## Supporting information

Supplementary Table S1.
