# Supplementary material for: Molecular profiling of ETS and non‐ETS aberrations in prostate cancer patients from northern India
Source: Prostate. 2015 Mar 23;75(10):1051–62. doi: 10.1002/pros.22989 (PMC4832366; doi:10.1002/pros.22989)
Supplement: Supplementary file 1 — Supplementary Table S1. [file PROS-75-1051-s001.doc]

| **SERIAL NO.** | **GLEASON SCORE** | **ERG STATUS** | **SPINK1**  **STATUS** | **PTEN STATUS** | **PATIENT’S**  **AGE** | **SURGERY**  **TYPE** |
| --- | --- | --- | --- | --- | --- | --- |
|  | 4+5 | POSITIVE |  | Homo | 76 | C |
|  | 4+3 | POSITIVE |  | Homo | 65 | T |
|  | 4+5 | POSITIVE |  | Homo | 85 | T |
|  | 4+5 | POSITIVE |  | Homo | 85 | T |
|  | 4+3 | POSITIVE |  | Homo | 63 | R |
|  | 4+5 | POSITIVE |  | Homo | 76 | C |
|  | NA | POSITIVE |  | Homo | 80 | C |
|  | 4+5 | POSITIVE |  | Homo | 85 | C |
|  | 4+3 | POSITIVE |  | Hetero | 60 | T |
|  | 4+3 | POSITIVE |  | Hetero | 60 | T |
|  | 4+3 | POSITIVE |  | Hetero | 70 | R |
|  | 4+5 | POSITIVE |  | Hetero | 65 | R |
|  | 4+5 | POSITIVE |  | Hetero | 72 | C |
|  | 3+4 (tertiary Gleason pattern 5) | POSITIVE | NEGATIVE | Hetero | NA^#^ | R |
|  | 4+3 | POSITIVE |  | Normal | 60 | C |
|  | 5+4 | POSITIVE |  | Normal | 80 | C |
|  | 3+4 | POSITIVE |  | Normal | 62 | C |
|  | 3+4 | POSITIVE |  | Normal | NA^#^ | C |
|  | 4+4 | POSITIVE |  | Normal | 82 | C |
|  | 4+3 (tertiary Gleason pattern 5) | POSITIVE |  | Normal | 60 | R |
|  | 4+3 | POSITIVE | NEGATIVE | Normal | 76 | T |
|  | 3+3 | POSITIVE |  | Normal | 53 | C |
|  | 5+4 | POSITIVE |  | Normal^$^ | 68 | C |
|  | 4+4 | POSITIVE |  | Normal | 70 | R |
|  | 3+3 | POSITIVE |  | Normal | 65 | R |
|  | 4+3 | POSITIVE |  | Normal | 70 | R |
|  | 3+4 | POSITIVE |  | Normal | 65 | R |
|  | 3+4 | POSITIVE |  | Normal | 72 | R |
|  | 3+4 | POSITIVE |  | Normal | 64 | R |
|  | 4+3 | POSITIVE |  | Normal | 62 | R |
|  | 3+3 | POSITIVE |  | Normal | 59 | R |
|  | 3+4 | POSITIVE |  | Normal | 64 | R |
|  | 3+4 | POSITIVE |  | Normal^$^ | 60 | R |
|  | 3+4 | POSITIVE |  | Normal | 65 | R |
|  | 4+3 | POSITIVE |  | Normal | 60 | C |
|  | 3+4 | POSITIVE |  | Normal | 57 | R |
|  | 3+4 | POSITIVE |  | Normal | 75 | C |
|  | 4+3 | POSITIVE |  | Normal | 71 | C |
|  | 3+4 | POSITIVE |  | Normal | 70 | C |
|  | 4+3 | POSITIVE | NEGATIVE | Normal | 68 | C |
|  | 4+5  **Supplementary Table: 1.** Clinicopathological characteristics of the prostate cancer specimens with *ERG* rearrangement,  *PTEN* loss and SPINK1 overexpression status. | POSITIVE |  |  | 68 | C |
|  | 3+3 | POSITIVE |  |  | 80 | C |
|  | 4+5 | POSITIVE |  |  | 60 | C |
|  | 4+3 | POSITIVE |  |  | 43 | R |
|  | 4+3 | POSITIVE |  |  | 70 | C |
|  | 3+4 | POSITIVE |  | NA | 79 | C |
|  | 4+3 | NEGATIVE | POSITIVE | Hetero | 66 | T |
|  | 3+4 | NEGATIVE | POSITIVE | Normal | 69 | R |
|  | 3+4 | NEGATIVE | POSITIVE | Normal | 66 | R |
|  | 4+3 | NEGATIVE | POSITIVE | Normal | 65 | R |
|  | 3+4 | NEGATIVE | POSITIVE | Normal^$^ | 57 | R |
|  | 3+3 | NEGATIVE | POSITIVE | Normal^$^ | 65 | R |
|  | 4+4 | NEGATIVE | POSITIVE | Normal^$^ | 65 | C |
|  | 4+3 | NEGATIVE | POSITIVE | Normal | 77 | R |
|  | 3+4 | NEGATIVE | POSITIVE | Normal^$^ | 75 | C |
|  | 3+3 | NEGATIVE | POSITIVE | Normal | 82 | C |
|  | 4+3 | NEGATIVE | POSITIVE | NA | 55 | T |
|  | 3+4 | NEGATIVE | POSITIVE | NA | 63 | R |
|  | 4+3 | NEGATIVE | NA | Homo | 66 | C |
|  | 5+4 | NEGATIVE | NEGATIVE | Hetero | 80 | C |
|  | 4+5 | NEGATIVE | NEGATIVE | Normal | 83 | C |
|  | NA | NEGATIVE | NEGATIVE | Normal | 75 | C |
|  | 4+3 | NEGATIVE | NEGATIVE | Normal | 64 | C |
|  | 4+3 | NEGATIVE | NEGATIVE | Normal | 88 | C |
|  | 4+5 | NEGATIVE | NEGATIVE | Normal^$^ | 72 | R |
|  | 4+3 | NEGATIVE | NEGATIVE | Normal | NA^#^ | T |
|  | 4+3 | NEGATIVE | NEGATIVE | Normal | 72 | R |
|  | 4+3 | NEGATIVE | NEGATIVE | Normal | 56 | R |
|  | 3+4 | NEGATIVE | NEGATIVE | Normal^$^ | 62 | R |
|  | 4+5 | NEGATIVE | NEGATIVE | Normal^$^ | 67 | R |
|  | 3+4 | NEGATIVE | NEGATIVE | Normal | 60 | R |
|  | 3+3 | NEGATIVE | NEGATIVE | Normal | 60 | R |
|  | 5+4 | NEGATIVE | NEGATIVE | Normal | 28 | C |
|  | 4+3 | NEGATIVE | NEGATIVE | Normal^$^ | 76 | C |
|  | 3+4 | NEGATIVE | NEGATIVE | Normal | 72 | C |
|  | 3+4 | NEGATIVE | NEGATIVE | Normal | 74 | C |
|  | NA | NEGATIVE | NA | Normal | NA^#^ | T |
|  | 3+4 | NEGATIVE | NA | Normal | 67 | T |
|  | 4+3 | NEGATIVE | NEGATIVE |  | 70 | C |
|  | Favor small cell/ Neuroendocrine | NEGATIVE | NEGATIVE | NA | 64 | C |
|  | 3+3 | NEGATIVE | NEGATIVE |  | 72 | C |
|  | 4+5 | NEGATIVE | NEGATIVE |  | 50 | C |
|  | 4+3 | NEGATIVE | NEGATIVE |  | 75 | C |
|  | 5+4 | NEGATIVE | NEGATIVE | NA | 52 | C |
|  | 4+3 | NEGATIVE | NEGATIVE |  | 58 | R |
|  | 4+4 | NEGATIVE | NEGATIVE |  | 44 | C |
|  | 4+4 | NEGATIVE | NEGATIVE | NA | 65 | C |
|  | 4+3 | NEGATIVE | NA |  | 72 | C |
|  | 3+4 | NEGATIVE | NEGATIVE |  | 60 | C |
|  | 4+3 | NEGATIVE | NEGATIVE |  | 50 | R |
|  | 4+4 | NEGATIVE |  | NA | NA^#^ | T |
|  | 4+4 | NEGATIVE |  | NA | NA^#^ | T |
|  | 4+4 | NEGATIVE |  | NA | NA^#^ | T |
|  | 4+3 (tertiary Gleason pattern 5) | NEGATIVE | NEGATIVE |  | 64 | R |
| **PCa Specimens Excluded from the Study** | | | | | | |
| 95 | NA | NA | NA | NA | 43 | R |
| 96 | NA | NA | NA | Normal | 56 | R |
| 97 | NA | NA | NA | Normal | 52 | R |
| 98 | NA | NA | NA | NA | 60 | R |
| 99 | NA | NA | NA | Normal | 58 | R |
| 101 | NA | NA | NA | Normal | 66 | R |
| 102 | NA | NA | NA | Hetero | 72 | C |
| 103 | NA | NA | NA | NA | 67 | R |
| 104 | 3+4 | NA | NA | Normal | 67 | C |
| 105 | 4+4 | NA | NA | NA | NA^#^ | T |
| 106 | 4+3 | NA | NA | NA | NA^#^ | T |
| 107 | 4+4 | NA | NA | Normal^$^ | 65 | T |
| 108 | NA | NA | NA | NA | 65 | T |
| 109 | NA | NA | NA | NA | 50 | C |
| 110 | NA | NA | NA | NA | 78 | C |
| 111 | 3+3 | NA | NA | NA | 76 | C |
| 112 | 5+4 | NA | NA | Hetero | 80 | C |
| 113 | NA | NA | NA | Normal | 75 | T |
| 114 | 3+3 | NA | NA | Normal | 82 | C |
| 115 | 4+3 | NA | NA | Normal | 64 | C |
| 116 | 4+4 | NA | NA | NA | 65 | C |
| 117 | 4+3 | NA | NA | NA | 88 | C |
| 118 | NA | NA | NA | NA | 43 | R |
| 119 | NA | NA | NA | NA | 56 | R |
| 120 | NA | NA | NA | NA | 52 | R |
| 121 | NA | NA | NA | NA | 60 | R |
| **NOTE:** **R**- Radical prostatectomy; **C**-TRUS guided core biopsy; **T**-Transurethral resection of prostate.  **Homo**- Homozygous; **Hetero**-Heterozygous. **NA**-Not Available.  **Normal^$^ -** Aneuploidy (> 2copies of Chr 10 & PTEN). **NA^#^** - Actual age not known; possibly between range of 65-75 years. | | | | | | |
